# Predicting a Rapid Transition to Telehealth-Delivered Parent–Child Interaction Therapy Amid COVID-19: A Mixed Methods Study

**DOI:** 10.1007/s43477-022-00057-0

**Published:** 2022-09-09

**Authors:** Yessica Green Rosas, Marika Sigal, Alayna Park, Miya L. Barnett

**Affiliations:** 1 Department of Counseling, Clinical, and School Psychology, Gevirtz Graduate School of Education, University of California, Santa Barbara, Santa Barbara, CA 93106-9490 USA; 2 Department of Human Ecology, University of California, Davis, Davis, CA USA; 3 Department of Psychology, University of Oregon, Eugene, OR USA

**Keywords:** Parent–Child Interaction Therapy, iPCIT, COVID-19, Telehealth, Implementation

## Abstract

The sudden onset of COVID-19 forced mental health therapists to rapidly transition to telehealth services. While some therapists and organizations were able to achieve an expeditious transition, others struggled. Using the Exploration, Preparation, Implementation, and Sustainment (EPIS) framework, which outlines key phases that guide the implementation process, the current mixed methods study examined what factors predicted the transition to internet-based Parent–Child Interaction Therapy (iPCIT), a telehealth-delivered evidence-based practice (EBP). We investigated two areas related to the transition: (1) if PCIT therapists transitioned to provide iPCIT and (2) if they made this transition quickly. In Fall 2019, 324 therapists completed a survey about implementing PCIT. After stay-at-home orders, 223 of those therapists completed a follow-up survey about their transition to telehealth, organizational characteristics, their caseloads, and telehealth training. The majority of therapists (82%) transitioned to provide iPCIT, with 48% making the transition in less than a week. Open-ended responses indicated that therapists who did not transition-faced challenges related to limited client resources, a lack of training, and organizational delays. Qualitative findings informed predictors for two logistic regression models that are statistical models that predict the probability of an event occurring, with criterion variables (1) whether therapists transitioned to provide iPCIT and (2) whether they transitioned in less than a week. Results showed that caseload in Fall 2019 and receipt of iPCIT training were associated with iPCIT transition. Organizational setting, resiliency, and baseline caseload predicted rapid transition to iPCIT. Implications regarding supporting the implementation of telehealth delivery of EBPs are discussed.

## Introduction

The COVID-19 pandemic introduced unique challenges for children, families, and mental health therapists worldwide. For example, parents experienced heightened levels of stress due to worries about virus exposure, increased job, and financial insecurity, as well as challenges working from home, online school, and a lack of childcare (Brown et al., 2020; Ganson et al., 2021; Haliwa et al., 2021; Thorell et al., 2022). Negative consequences of heightened stress during the pandemic have affected both parents as well as children. This can be seen in elevated levels of maternal anxiety and depression, as well as an increased risk of parental burnout and child maltreatment (Cameron et al., 2020; Griffith, 2020; Imran et al., 2020). Recent research findings suggest that parents’ emotion regulation skills are associated with child stress responses and can mediate negative effects of child exposure to stressful life events (Ganson et al., 2021). Thus, access to high-quality evidence-based services was especially important for parents experiencing elevated levels of stress during the COVID-19 pandemic.

Many therapists’ in-person services were suddenly interrupted due to COVID-19-related stay-at-home mandates, and a rapid transition to a telehealth model was necessary to ensure continued delivery of treatment. While some therapists were able to rapidly make this transition, others struggled amid sudden changing guidelines and procedures. Karayianni et al. (2021) posed that evidence-based practices (EBPs) were especially challenging to implement during a pandemic, in part due to limited data and funding available. Past research has reported on challenges in delivering EBPs via telehealth, including clinical procedures requiring higher levels of preparation, slight changes to treatment protocols, as well as technological considerations, while simultaneously acknowledging the potential benefits that telehealth can have in disseminating EBPs for traditionally underserved populations (Gros et al., 2013). Indeed, despite the challenges posed, efforts have been made to continue the delivery of EBPs for children and families in need during COVID-19 restrictions. This paper specifically investigates the transition to deliver Parent–Child Interaction Therapy (PCIT) via telehealth. PCIT is an EBP originally developed to address disruptive behaviors, which has been extended to effectively address a range of child emotional problems (i.e., anxiety and depression), along with child physical maltreatment (Lieneman et al., 2017; Thomas & Herschell, 2013). PCIT was identified as being a strong fit to deliver during COVID-19 as it addressed many of the pressing parenting and child challenges families were facing and had previously been adapted for telehealth delivery (Gurwitch et al., 2020).

### Applying EPIS to the Transition to Telehealth During COVID-19

Implementation science offers various frameworks that help to understand the conditions necessary to support the uptake of new innovations and evidence-based practices. The Exploration, Preparation, Implementation, and Sustainment (EPIS; Aarons et al., 2011; Moullin et al., 2019) model provides a strong fit to understand different processes needed to successfully implement EBPs in the context of the COVID-19 pandemic. The EPIS model describes how outer systems (e.g., policy) and inner contexts (e.g., agency leadership, therapist characteristics), along with innovation (e.g., EBP characteristics) and bridging factors (e.g., purveyor organizations) impact implementation processes (Moullin et al., 2019).

### Outer Systems Factors

During the COVID-19 pandemic, a variety of policy changes were made quickly to limit the spread of the virus, including increased coverage for telehealth visits and an emergency order by the federal government whereby therapists were protected against penalty for any unintended HIPAA violation under the good faith provision of telehealth (U.S. Department of Health and Human Services, 2020). Beyond these outer context factors, additional research is needed to identify inner context factors, innovation characteristics, and bridging factors that support rapid transitions in the face of crises, such as the COVID-19 pandemic.

### Inner Context Factors

Inner context factors, such as organizational characteristics, may be of particular importance when examining the transition to evidence-based telehealth services during COVID-19. For example, organizational climate, which is the shared perceptions of the psychological impact of the work environment on the therapist, has been thought to affect both the adoption of EBPs as well as the quality of delivered services (Glisson & James, 2002). Within implementation science, various aspects of organizational culture and climate have been investigated, with a focus on aspects that lead to the adoption and sustainment of EBPs (Glisson, 2007; Weiner et al., 2011). During the onset of the COVID-19 pandemic, organizations needed to decide how to rapidly adopt a telehealth treatment model in order to keep their employees and the families that they served safe. The ability for organizations to respond to uncertainty and crisis successfully is referred to as organizational resilience (Lee et al., 2013). Although this construct has not been widely studied within implementation science, the context of COVID-19 points to the importance of understanding how organizations were able to quickly pivot in their service delivery and support the continuation of high-quality, evidence-based care.

Another inner context factor that likely impacted the transition to telehealth relates to the clients served by an organization. Given that remote services do not require clients to travel to a treatment facility, conducting telehealth sessions can make treatment more accessible for clients who have busy schedules and/or issues with transportation. However, technological concerns arise given that not all families may have the equipment necessary to engage in remote treatment (e.g., having a laptop or tablet and consistent access to high-quality internet), along with digital literacy (Beaunoyer et al., 2020; Khilnani et al., 2020). Indeed, concerns have been raised regarding the need to actively avoid any potential exacerbation of mental health service inequalities during COVID-19 for families who face structural barriers to telehealth services (Moreno et al., 2020). Furthermore, client fit and comfortability with telehealth services may also impact delivery of treatment. For example, challenges have been reported in engaging children when conducting remote sessions (Sklar et al., 2021).

### Innovation Characteristics

Regarding innovation, PCIT provides a unique evidence-based practice to study within the context of the widespread transition to telehealth spurred by COVID-19. Internet-Based PCIT (iPCIT) had an emerging evidence-base before the 2019 pandemic (Comer et al., 2017; Gurwitch et al., 2020). PCIT includes two phases: the child-directed interaction (CDI) phase, which focuses on enhancing the parent–child relationship through play based skills, and the parent-directed interaction (PDI) phase, which teaches parents effective discipline (Comer et al., 2017). Core components of PCIT are retained in iPCIT. These core components include an emphasis on the parent–child relationship, the use of in vivo feedback (i.e., coaching) to modify parent behaviors and administration of weekly assessments of parent skill use and child behavior problems. In iPCIT, the parent receives in vivo coaching via either a Bluetooth device directly connected to a visual interface (i.e., computer, tablet, or cellphone screen) or headphones connected separately to a cellphone. Studies comparing traditional in-person PCIT with iPCIT have demonstrated promising results for iPCIT (Comer et al., 2017; Kohlhoff et al., 2019). In a randomized control trial, children who received iPCIT were more likely than those who received standard PCIT to be rated by evaluators masked to treatment condition to have an “excellent response” at post-treatment and the 6-month follow-up (Comer et al., 2017). Furthermore, results indicated significantly fewer parent-perceived barriers to treatment for iPCIT in comparison to clinic-based PCIT, as well as high-treatment satisfaction ratings for both iPCIT- and clinic-based delivery. Although iPCIT has evidenced promising outcomes, it had not been widely implemented prior to COVID-19 in part because of the challenges that arise with insurance (e.g., billing and reimbursement) as well as variability in technological capacity for clients and organizations (Gurwitch et al., 2020).

### Bridging Factors

Bridging factors include processes that connect inner organizational contexts and outer systems. (Lengnick-Hall et al., 2021). During the COVID-19 pandemic, PCIT purveyor organizations, which oversee training and certification in the model, made efforts to offer resources regarding how to deliver iPCIT. These resources were made available for free and disseminated via their listservs of therapists (http://www.pcit.org/covid-19-professional-resources.html). More literature is needed to better understand how these efforts were drivers of a rapid transition to telehealth-delivered services.

### Present Study

The current study sought to examine which organizational, client, and implementation support characteristics predicted rapid transition to delivering iPCIT during COVID-19. The study team included five graduate students who coordinated various aspects of the research project, including data collection and management, as well as managing coding teams and four undergraduate-level research assistants who primarily supported in coding qualitative responses. All members of the team were supervised by a faculty-level principal investigator. In Fall 2019, 324 PCIT therapists completed a survey about PCIT implementation. We followed up with these therapists in Summer 2020 to investigate whether these therapists transitioned to provide iPCIT and to explore barriers and facilitators that might have impacted their transition. Specifically, the current study employed a mixed methods design to (1) quantitatively describe the percentage of therapists who transitioned to deliver iPCIT, (2) qualitatively identify barriers and facilitators to transitioning to iPCIT, and (3) quantitatively examine the association between the identified iPCIT barriers and facilitators and rapid transition to implementing iPCIT.

## Methods

### Procedure

The participants in this study were therapists (*N* = 324) originally recruited via listservs run by PCIT training organizations (i.e., PCIT International, UC Davis) in Fall 2019 for a study assessing PCIT implementation. In Summer 2020, a follow-up survey invitation was sent to a total of 309 therapists who had provided their contact information at baseline in order to see how they had adapted their services during the COVID-19 pandemic. Of the 309 therapists contacted, 223 therapists (72%) completed the survey regarding their delivery of PCIT during COVID-19. Therapists were sent a unique Qualtrics link to allow their responses to be linked with their baseline survey. At the beginning of the survey, therapists were asked questions about whether they transitioned to delivering PCIT via telehealth during COVID-19. Qualtrics display logic was used to display the next set of appropriate question based on participants’ previous answers. Therapists who reported transitioning to telehealth were asked qualitative and quantitative questions regarding iPCIT implementation. Therapists who indicated that they did not transition to iPCIT answered questions about barriers and facilitators to transitioning to iPCIT and reasons for not transitioning to iPCIT (e.g., organization, client, personal). After completing the survey, therapists were e-mailed a $20.00 Amazon gift card for their participation. This study was determined exempt by the Institutional Review Board at [masked for review].

### Participants

Participants were predominately female (90%), with an average therapist age of 36.42 years (*SD* = 8.24). Majority of therapists self-identified as non-Hispanic, White (74%), and were certified in PCIT (70%). Half of our sample (50%) worked in a community-based clinic and more than a quarter described their discipline as clinical psychology (35%). A large proportion of therapists (89%) indicated that they were working at the same agency they were working for during the first survey in 2019. Regarding reimbursement for services, 39% of therapists saw predominately clients with Medicaid or state insurance, 14% saw predominately private pay clients, 13% saw predominately clients with private insurance, and 2% saw predominately uninsured clients. Participants had an average of 18 clients on their caseload, including 5 PCIT clients on average. Additional sample characteristics can be found in Table 1.

**Table 1:** Sample characteristics

	Total^a^	Transitioned^b^	Did Not Transition^c^
Therapists’ age M (SD)	36.42 (8.24)	36.80 (8.44)	34.65 (7.09)
Therapists’ gender			
Female	89.7%	89.6%	90.0%
Male	9.9%	9.8%	10.0%
Non-binary/gender queer	0.4%	0.5%	0.0%
Therapists’ ethnicity			
Latinx	17.0%	15.8%	22.5%
Non-Latinx	83.0%	84.2%	77.5%
Therapists’ race			
White	86.0%	87.0%	81.6%
Black/African American	2.8%	2.8%	2.6%
Asian/Pacific Islander	3.3%	4.0%	0.0%
American Indian/Alaska Native	0.9%	0.0%	5.3%
Multiracial	3.3%	3.4%	2.6%
Other	3.7%	2.8%	7.9%
Therapists’ mental health discipline			
Clinical psychology	35.4%	37.7%	25.0%
Marriage family therapy	21.1%	21.3%	20.0%
Counseling	21.1%	18.6%	32.5%
Social work	18.8%	19.1%	17.5%
School psychology	2.2%	2.2%	2.5%
Psychiatry	0.4%	0.5%	0.0%
Other	0.9%	0.5%	2.5%
Therapists’ primary work setting			
Community-based clinic	50.0%	50.3%	48.4%
Academic institution	26.5%	27.9%	19.4%
Private practice	23.5%	21.8%	32.3%
Implementation strategies M%^d^			
Training materials about general telehealth	66%	67%	60%
Consultations from within the agency	57%	61%	38%
Training materials about iPCIT	54%	57%	40%
Webinar trainings from PCIT International	50%	55%	28%
Webinar trainings from outside agencies (e.g., APA)	47%	51%	30%
Consultations from outside the agency	27%	27%	30%
Live observation/feedback of telehealth sessions	18%	19%	15%
Reviewing telehealth cases	13%	13%	15%
None	4%	2%	10%

^a^Total sample *n* = 223

^b^Transitioned to iPCIT *n* = 183

^c^Did not transition to iPCIT *n* = 40

^d^Mean proportions calculated from total selected trainings

Differences in therapist demographics were compared for individuals who participated only in the original survey to those who completed the follow-up survey related to COVID-19, with no differences identified for therapist gender, X^2^ (2) = 3.78, *p* = 0.151; race, X^2^ (5) = 3.89, *p* = 0.566; ethnicity, X^2^ (2) = 0.042, *p* = 0.979; age, *t*(321) = 1.59, *p* = 0.114; number of total cases, *t*(147) = 1.36, *p* = 0.175; nor PCIT cases, *t*(321) = 0.814, *p* = 0.416. As there were no significant differences between participants who responded to the follow-up survey and those who did not, it appeared to be a representative sample of therapists from the original study.

### Measures

#### Therapist Characteristics

The Therapist Background Questionnaire (Brookman-Frazee et al., 2012) was modified to collect demographic information at baseline of therapists’ personal characteristics (e.g., age, gender, ethnicity), education, work experience (e.g., type of services provided), and agency type (i.e., community-based clinic, academic setting, private practice). In the follow-up survey, therapists were also asked if they still worked at the same agency as baseline. Additional therapist characteristics are included in Table 1.

#### Caseload Characteristics

Therapists were asked to report their current total and PCIT caseload size at baseline and in the follow-up survey. To gain a better understanding of the individuals being served by therapists, information on racial/ethnic composition of clients and insurance (i.e., uninsured, private pay, Medicaid/state insurance, private insurance) was also collected at both time points.

#### Organizational Resilience

Therapists rated their organization’s resilience and preparedness for emergencies using a 13-item Benchmark Resilience Tool Short Form B (BRT-13B; Whitman et al., 2013). The BRT-13B assesses an organization’s ability to plan (e.g., *We believe emergency plans must be practiced and tested to be effective*) and adapt (e.g., *Our organization can make tough decisions quickly*). Response choices were on a Likert scale ranging from “Strongly Disagree” (1) to “Strongly Agree” (4), with higher scores indicating higher organizational resilience. Responses were averaged to create a composite score. Cronbach’s alpha for this sample was 0.87.

#### Implementation Strategies

Based on research on implementing iPCIT during COVID-19, we had therapists select the implementation strategies they used regarding delivering iPCIT and general telehealth (Garcia et al., 2021a, 2021b). Specifically, therapists were asked, “What training did you participate in relating to telehealth? (Select all that apply).” Therapists were given two PCIT-specific training response options, six general telehealth training options, an option to select “Other” and an option to select “None.” Open-ended responses for “Other” were reviewed and manually coded into iPCIT supports because all these responses were related to iPCIT. The proportion of therapists who selected each training option can be found in Table 1. For the logistic regression analyses, the response choices that were specific to iPCIT training (e.g., “Webinar trainings from PCIT International”) and those that were related to general telehealth (e.g., “Training materials about general telehealth”) were summed and computed into two new variables (i.e., iPCIT supports and general telehealth supports) and entered into the models.

#### Barriers and Facilitators to iPCIT Transition

To identify what barriers hindered iPCIT transition and what facilitators would have promoted this transition, only therapists who responded that they did *not* provide iPCIT during COVID-19 were asked the following open-ended items: (1) What supports would facilitate providing PCIT via telehealth? And (2) Please elaborate about the challenges to transitioning to PCIT via telehealth.

### Data Analytic Plan

A mixed method design was used for the current study, in which qualitative and quantitative online survey data were simultaneously collected (Palinkas et al., 2011). We then employed a sequential QUAL + QUAN (i.e., qualitative findings informing quantitative investigation) analysis to examine factors associated with the rapid transition to iPCIT during COVID-19. Qualitative themes regarding barriers transitioning to iPCIT informed the predictors that were entered into the quantitative models. The function of the mixed method approach was triangulation (i.e., using different methods to inform the same research question), to see if both types of methods (qualitative and quantitative) identified similar factors impacting the rapid transition to iPCIT during COVID-19.

### Qualitative Analyses

Of the 40 therapists who did not provide iPCIT during COVID-19, 23 expanded on transitional barriers and 25 detailed possible facilitators. Open-ended responses were coded following recommendations for conducting qualitative analyses within mental health services and implementation research (National Cancer Institute, 2018; Palinkas et al., 2011). First, a coding team of three coders (two graduate student research assistants and one undergraduate research assistant) read all responses and developed a coding manual with 3 umbrella themes (i.e., therapist related, client related, organization related), 10 subthemes for barriers to transition, and 5 subthemes for facilitators to transition. Research assistants used the coding manual to individually code the first 25% of responses. Next, group consensus on occurrence or non-occurrence of codes was reached to ensure the reliability of the coding manual’s definitions before research assistants moved forward with the next half of the responses. This method continued in a stepwise manner until all responses were coded and consensus was reached on each open-ended response. Following coding, the entire authorship team reviewed and finalized themes.

#### Quantitative Analyses

Two logistic regression models were run to predict (1) whether therapists transitioned to deliver iPCIT and (2) whether they did so rapidly, operationalized as less than a week. Based on qualitative analyses (described below in the results section), the following predictors were included in each model: organization type (i.e., academic institution, private practice, community-based clinic), perceived organizational resilience, baseline PCIT caseload, types of telehealth training received (i.e., iPCIT specific and general telehealth), and proportion of PCIT caseload who were uninsured or on Medicaid. Additionally, we controlled for if participants changed organizations between the two surveys. Quantitative analyses were conducted using SPSS 28, a software used to manage data and run statistical analyses.

## Results

### Therapists’ Characteristics

Of the 223 therapists who completed our follow-up survey, 82% reported that they transitioned to deliver iPCIT and 48% did so in less than a week. Thirty-one percent of therapists reported taking one to two weeks, 18% took two weeks to a month, and 3% took over a month. Notably, almost all therapists in our sample accessed telehealth trainings and only 8 therapists indicated that they did not access any general or iPCIT-specific implementation strategies during COVID-19. Both therapists who transitioned and therapists who did not transition reported utilizing training materials about general telehealth and consulting within their agency the most. The full list of implementation strategies and subsample frequencies can be found in Table 1.

### Barriers to iPCIT Transition

Of the 40 therapists who did not provide iPCIT, 34 therapists identified barriers that made the transition difficult. Ten therapists were excluded for indicating the question was not applicable to them (i.e., responded with “N/A” or “not providing clinical services”) and one therapist was excluded for not experiencing any challenges providing iPCIT. Final thematic coding was conducted on 23 responses.

Therapists reported experiencing administrative and logistical barriers based on the need to make a rapid transition to iPCIT in response to COVID-19 and stay-at-home orders. For instance, some therapists (*n* = 5) reported organization-specific barriers to iPCIT transition, “My agency was not prepared [for] [COVID-19], and services are on hold until they figure out a plan.” Additionally, therapists reported having concerns with billing, as well as their organizations lacking HIPAA compliant software and appropriate technological equipment. Further, there was a notable decrease in PCIT referrals as one therapist explained, “We have a low number of referrals right now due to COVID-19 […].” COVID-19 may have disproportionally affected organizations depending on their primary source of client referrals as another therapist reported, “The biggest challenge has been referrals for our agency. Majority of our referrals for PCIT come from the school system and hospitals.”

A lack of training in iPCIT was also a barrier for a few therapists in our sample. One therapist said their biggest challenge to providing iPCIT was “a lack of knowledge on how to actually do it.” Another therapist reported observing “staff hesitancy [to provide iPCIT] due to a lack of training […].” Therapists also experienced personal barriers to providing iPCIT. One therapist reported lacking confidence in their ability to do iPCIT, as well as experiencing “stress in dealing with pandemic.” Another therapist reported experiencing logistical barriers to implementing iPCIT due to being deaf, “I am deaf/with a cochlear implant and have difficulty hearing over zoom/facetime/other video conferencing services and over the phone unless I have direct access to the speaker’s face to be able to lip-read.” Transitioning to iPCIT may have been especially difficult for therapists with specific accommodations as this therapist continued to explain, “This has complicated my ability to provide PCIT in a way that would be most similar to how I provide it in-person.”

Although organizational and personal barriers were experienced by many therapists, the most mentioned barriers were related to clients (*n* = 14). Namely, “families not having the equipment or feeling comfortable” were the most common issues experienced with clients. Therapists identified concerns about space and time also limited willingness to do iPCIT. Some clients could not attend iPCIT sessions during COVID-19 due to personal obligations as one therapist explained, “The families I was working with no longer had childcare for other children to provide the necessary time to devote to session.” Distractions in the home such as other children or family members also hindered engagement during sessions, as some therapists stated.

Multiple therapists (*n* = 7) reported that clients refused to give iPCIT a try and instead “[…] wanted to do PCIT in the office.” One therapist said they wanted to provide iPCIT but could not due to client refusal, “My clients have been very resistant or else I would be doing it.” Therapist responses pointed to the need for additional strategies to recruit appropriate families for PCIT during COVID-19. To improve PCIT caseload numbers, one therapist in our sample suggested “community marketing to bring more referrals […].” Another therapist suggested marketing past client experiences to potential new clients, “Testimonials from [PCIT] clients that current clients could see about doing [iPCIT].”

A lack of technological resources and technical difficulties were the second most mentioned client-related barrier to iPCIT transition (*n* = 6). One therapist stated that “families [not] having the correct equipment” was a significant barrier to providing iPCIT. Another therapist explained that families lacked wireless accessories which were logistically better suited for iPCIT sessions, “[…] having tech for the wireless headphones/ear buds which was more ideal.” When families did have the right tools for the session, frequent technical issues prevented clients and therapists from having a smooth iPCIT experience, “Audio problems; some families have difficulty with good internet connection and navigating telehealth platforms.” Moreover, therapists with low-income clients felt the most need for resources and technological assistance as one therapist explained, “Families participating in PCIT not having internet access or even minutes on their cell phones.” Technological issues also exasperated difficulties with the most stressful parts of PCIT treatment, such as the Parent-Directed Interaction (PDI) phase. One therapist explained the lack of therapist control in this phase during iPCIT, “You have less control of having a time out room and chair that works well in the home—depending on the family’s available resources.” Another therapist noted client difficulty with “PDI procedure, without having practiced in the clinic first.”

### Facilitators of iPCIT Transition

Thirty-one therapists responded to the open-ended question asking what facilitators would promote iPCIT transition. Six respondents were excluded because they were not sure what facilitators would help (i.e., “I don’t know” or “Not sure”), they did not specify any facilitators (i.e., “Current resources are useful”) or the question was not applicable to them (i.e., “N/A no longer providing clinical services”). A total of 25 responses were thematically coded for this open-ended item.

The most mentioned resources needed to improve iPCIT transition by a large majority were technology related (*n* = 14). Specifically, therapists shared an overwhelming need for electronic devices and accessories (e.g., wireless headsets, tablets) and access to high-speed internet for their clients. Several therapists suggested providing agency funding “to be able to provide PCIT toys/materials for families to use and keep for their sessions.”

The second most noted theme in therapists’ responses was related to training and mentoring (*n* = 10). Therapists asked for instructional demonstrations of iPCIT and/or mentoring from therapists who had iPCIT experience. One therapist thought “opportunities for co-therapy with seasoned therapists experienced with PCIT via telehealth” would be beneficial. Another therapist suggested “more webinars with case videos.” Support for “[…] PCIT trainers regarding how to provide remote live training/coaching” may promote iPCIT transition as well, as suggested by another therapist in our sample. A barrier to accessing trainings and resources may have been due to a lack of available free time, as some therapists in our sample felt that getting time off to seek trainings would facilitate iPCIT transition. Lastly, a few therapists addressed the need for additional staffing. Specifically, one therapist highlighted the need for “administrative staff who can provide technical assistance to the families.”

### Predicting iPCIT Transition

The following themes related to organizational, implementation support, and client-level factors identified through qualitative coding were entered into two logistic regression models predicting (1) therapist transition to delivering iPCIT and (2) therapist transition in less than a week. To address themes regarding organizational readiness, perceived organizational resiliency scores were entered into the model. Organization type (i.e., academic institution, private practice, community-based clinic) was also included to address differences in the types of resources and clientele served by these settings. Regarding challenges identified with referrals and client resources (i.e., access to internet, appropriate toys and adequate living space), PCIT caseload from survey one and proportion of PCIT caseload who were uninsured or on Medicaid were included in the model. Regarding iPCIT delivery potentially being facilitated by trainings and mentoring, therapist participation in trainings related to the provision of telehealth services and, more specifically, iPCIT were included in the model. Finally, we controlled for if participants changed organizations at follow-up given the impact of turnover on maintaining an EBP (Woltmann et al., 2008).

#### Transition to iPCIT

Logistic regression results showed that total number of PCIT clients at baseline and receipt of iPCIT-specific supports significantly predicted whether therapists transitioned to iPCIT during COVID-19. Specifically, the expected odds of a therapist transitioning to iPCIT increase by 1.23 for each additional PCIT client in their baseline caseload, *b* = 0.208, *SE* = 0.087, Wald χ^2^(1) = 5.774, *p* = 0.016. Furthermore, therapists were 3.04 times more likely to transition to iPCIT if they received iPCIT-specific supports, *b* = 1.110, *SE* = 0.448, Wald χ^2^(1) = 6.130, *p* = 0.013. No other organizational, implementation support, or client-level factors predicted whether therapists transitioned to iPCIT.

#### Transition in Less Than a Week

Logistic regression results also showed that total number of PCIT clients at baseline, organizational resilience (BRT-13B score), and agency setting predicted whether therapists transitioned to iPCIT in less than a week. Specifically, the expected odds of transitioning to iPCIT in less than a week increase by 1.09 for each additional PCIT client in the baseline caseload, *b* = 0.086, *SE* = 0.042, Wald χ^2^(1) = 4.297, *p* = 0.038. The expected odds of a therapist rapidly transitioning to iPCIT increase by 3.31 for each 1-point increase in BRT-13B score, *b* = 1.196, *SE* = 0.404, Wald χ^2^(1) = 8.790, *p* = 0.003. Additionally, therapists working in private practice were 5.16 times more likely to rapidly transition to iPCIT than therapists working in community-based clinics, *b* = 1.641, *SE* = 0.686, Wald χ^2^(1) = 5.723, *p* = 0.017. No other organizational, implementation support, or client-level factors predicted whether therapists transitioned to iPCIT in less than a week. The complete list of predictors for both models can be found in Table 2.

**Table 2:** Logistic regression models predicting time and transition to iPCIT

Predictors	Transition to iPCIT				Rapid Transition to iPCIT			
	*b*	SE	*p*	OR [95% CI]	*b*	SE	*p*	OR [95% CI]
Baseline PCIT caseload	.21	.09	.02*	1.231 [1.039–1.459]	.09	.04	.04*	1.090 [1.005–1.183]
Stayed at baseline agency	− 1.10	.66	.10	.334 [.092–1.209]	1.11	.78	.16	3.022 [.650–14.056]
Organizational resilience	.24	.47	.61	1.269 [.506–3.184]	1.20	.40	.00*	3.308 [1.500–7.296]
Academic setting v. community-based clinic	.62	.64	.33	1.862 [.529–6.551]	−.14	.48	.76	.867 [.342–2.201]
Private practice v. community-based clinic	.26	.72	.72	1.297 [.317–5.314]	1.64	.69	.02*	5.162 [1.345–19.803]
Medicaid/uninsured caseload	.01	.01	.30	1.007 [.994–1.020]	.01	.01	.17	1.008 [.997–1.019]
iPCIT-specific supports	1.11	.45	.01*	3.035 [1.260–7.308]	−.05	.44	.91	.952 [.401–2.259]
General telehealth supports	1.18	.62	.06	3.237 [.961–10.908]	−1.33	.77	.09	.265 [.059–1.203]

*N* = 223. Rapid transition = Less than 1 week following stay-at-home orders

*p* < .05*

## Discussion

Our study found that the vast majority of PCIT therapists transitioned to providing iPCIT, with 82% of our respondents reporting they had made the transition and 48% doing so in a week or less. These findings were encouraging, as they pointed to the sustainment of the EBP, PCIT, during a time of increased stress for children and parents. Consistent with the EPIS Framework, the implementation of iPCIT was likely facilitated by various outer context factors (e.g., policies related to insurance reimbursement for telehealth services) and innovation and bridging factors. Our study helped to illuminate how inner context factors predicted the transition to iPCIT, along with the role of specific training in the model.

Regarding innovation and bridging factors, the fact that PCIT had been previously adapted for telehealth facilitated purveyor organizations being able to quickly develop and disseminate training materials (Barnett et al., 2021a, 2021b; Gurwitch et al., 2020). A manual and video training with considerations for telehealth delivery of PCIT (e.g., how to set up the playroom for PCIT) were created and provided for free on PCIT listservs and websites. This was especially important as receiving iPCIT-specific training supports predicted iPCIT implementation during COVID-19, whereas general trainings in telehealth did not. These findings extend on the importance of PCIT purveyor organizations making training in telehealth delivery of the specific intervention available, as a previous study found that getting specific training in delivering iPCIT was associated with improved child and parent outcomes, whereas general training in telehealth was not (Garcia et al., 2021a, 2021b). Maintaining listservs for trained therapists might help promote sustained implementation during times of crises, in that information about adaptations, additional trainings, and strategies to address world events can be quickly disseminated. Indeed, 72% of therapists reported that they had accessed trainings specific to iPCIT, whereas therapists who did not transition to iPCIT identified that a lack of training in the model was a barrier to their use of the intervention. Tailoring implementation supports to meet therapist confidence in telehealth models of EBPs might be necessary, as most but not all therapists in our sample were able to access the online trainings and manuals made available by PCIT purveyor organizations. Additional local and hands on support, such as supervision and training within the therapist’s own organization, were likely important for some therapists with less confidence in telehealth.

Having a larger caseload in Fall 2019 predicted both the likelihood that therapists transitioned to iPCIT and the rapid transition time. Having a larger PCIT caseload has been associated with better implementation outcomes, including higher skill levels following consultation calls and sustained delivery of the model over time (Barnett et al., 2021a, 2021b; Jackson et al., 2017). The higher caseload in Fall 2019 could indicate several factors related to transitioning to iPCIT in this study. First, therapists with a robust PCIT caseload may be more dedicated to implementing this EBP, which could motivate them to pursue additional training opportunities in iPCIT. Additionally, as PCIT caseloads were shown to decrease slightly following the transition to telehealth during COVID-19 (Barnett et al., 2021a), therapists with a larger caseload would be more likely to have more clients who could continue with care.

The importance of caseload and qualitative responses regarding the need for increased referrals point to the need for improved recruitment strategies, including direct-to-consumer marketing, to support PCIT implementation generally and iPCIT specifically. Although therapists in the study suggested that testimonials from parents might improve engagement, it is important to test the effectiveness of these strategies, especially for diverse groups. For example, one study on direct-to-consumer marketing actually found that Spanish-speaking parents had higher intentions in pursuing PCIT if the testimonial was delivered by a therapist as opposed to a parent (Barnett et al., 2020). Therefore, it is important to tailor recruitment strategies for different populations, with a focus on increasing equity in who is accessing care.

In qualitative responses, many therapists mentioned a lack of client resources (e.g., toys, electronic accessories, high-speed internet) as a barrier to transitioning to PCIT. Even though client insurance status was not associated with transitioning to iPCIT and previous research has shown that diverse families continued with iPCIT in the wake of COVID-19 (Barnett et al., 2021a, 2021b; Garcia et al., 2021a, 2021b), these qualitative findings are important to consider when identifying how telehealth services impact equity. This is especially relevant given that the negative impacts of COVID-19 fell disproportionately among minoritized groups (Louis-Jean et al., 2020). For example, the Centers for Disease Control and Prevention reported that Latinx individuals accounted for 25% of COVID-19 cases in the USA, despite only representing 18% of the population (CDC, 2020). Low-income families experienced compounded levels of stress due to limited household space and heightened financial insecurity, and youth of color were to be at a higher risk of mental health challenges during the pandemic (Surgeon General, 2021). Additionally, although telehealth has been proposed as a solution to increase access for rural populations, our qualitative findings point to challenges with the availability of high-speed internet access. These findings are consistent with research finding that rural psychologists were less likely to transition to telehealth during the COVID-19 pandemic (Pierce et al., 2021).

To relieve some of the financial burden and lessen participation anxiety of iPCIT, take-home kits (as suggested by one therapist in our sample) containing essential resources for iPCIT (e.g., low-costing Bluetooth headset, toy) for families who are low income and/or those who lack needed resources for sessions may be a helpful solution to promote equity. Additionally, given identified barriers due to the families’ comfort with technology, organizations may want to consider providing step-by-step instructional videos on how to setup and use telehealth software to clients, as well as having administrative staff specifically dedicated to technical support. Based on therapists’ feedback in this study, these strategies could significantly lessen the impact of technological barriers to iPCIT and help decrease the additional time burden for therapists. Although take-home kits and additional technology supports are a smart solution to enhance access, barriers related to infrastructure, such as availability and access to high-speed internet, are harder to overcome through organizational funding alone.

Organizational setting also related to the speed in which therapists transitioned to iPCIT. Therapists working at private practices were significantly more likely to transition to iPCIT in less than a week than community-based therapists, possibly due to the differences in resources between these two organization types. Private practice therapists may have already had training in and/or conducted telehealth therapy prior to the onset of COVID-19, which could have limited barriers to iPCIT transition for both private practice therapists and their clients. Further, private practice therapists and the clients they served may have had more financial resources to obtain electronic accessories needed to provide iPCIT. Conversely, therapists at community-based clinics may have had to delay transition due to bureaucratic barriers (e.g., putting policies into place for telehealth delivery) or a lack of resources. Notably, within our sample almost 80% of therapists reported making the transition in under two weeks, pointing to the speed in which this transition was made across the vast majority of therapists.

Various aspects of organizational climate and culture (e.g., implementation climate, organizational readiness for change) have been associated with the adoption and sustainment of EBPs (Ehrhart et al., 2014; Glisson, 2007; Weiner, 2009; Weiner et al., 2011); however, this study was unique in investigating the role of organizational resilience at providing an EBP via telehealth during a public health crisis. The finding that organizational resilience predicted the rapid transition to iPCIT in our sample points to the importance of having organizations develop strategies to be able to adapt and respond to emergent emergencies that might arise. Given increasing rates of climate-related disasters and civil conflict, it is critical to understand what allows for the continuity of critical mental health services during crises (Palinkas et al., 2020).

## Strengths and Limitations

This study has several strengths, including its use of mixed methods with a longitudinal sample to study how PCIT implementation was influenced by the COVID-19 pandemic and its consideration of organizational, implementation support, and client-level factors. However, there are some study limitations that warrant further discussion. First it is important to recognize that the quantitative analyses identified correlational associations with the transition to iPCIT, which were triangulated with qualitative data, but further research would be needed to establish the cause and effect of the predictors. Further, open-ended responses provided some qualitative data, but other forms of data collection (e.g., interviews or focus groups) would have allowed for follow-up questions to gain deeper insights into therapist perspectives on the challenges of transitioning to iPCIT. Although the current sample appeared to be representative of participants from the original sample and the response rate was high (72%), it is possible that this sample is not broadly representative of therapists that have been trained in PCIT. The majority of participants were certified in the model, which indicates they may have more commitment to delivering PCIT and sustaining its delivery during COVID-19 than therapists who did not complete either survey. Further, the sample of therapists had limited racial and ethnic diversity. Although this is consistent with national reports on the racial and ethnic diversity of mental health care providers generally and PCIT therapists specifically (Lin et al., 2018; Salsberg, et al., 2020, The Justice Collective, 2021), the demographics of our sample limited our ability to identify how COVID-19 impacted therapists of color. Finally, it is important to acknowledge that this study specifically investigated one EBP, and findings may not generalize to the factors that facilitated or limited the uptake of other EBPs during COVID-19.

## Implications and Future Directions

This study provides important insights about how to sustain EBPs during crises. This is especially pertinent as climate-related disasters and political unrest continue to require rapid responses and adaptations from service sectors (Palinkas et al., 2020). Although the COVID-19 pandemic was a unique worldwide event, these findings may help organizations and therapists prepare for future emergencies and sudden onset of challenges. One area highlighted in our findings is the importance of developing and testing strategies to promote organizational resilience as this may help organizations continue to provide EBPs in the face of emergent crises. It will be especially important for future research and practice to focus on how to promote equity in access to EBPs during crises, as many minoritized groups are likely to have disparities exacerbated by the trauma inflicted by the event while facing barriers to receiving high-quality mental health care services (Liu & Modir, 2020). Additionally, although telehealth may increase access for families in rural locations without service providers, increased availability of reliable high-speed internet is needed. Overall, COVID-19 led to rapid changes in mental healthcare delivery, which could have long-lasting consequences as to how services are provided. Hopefully, lessons from this pandemic spur systemic changes, such as improved internet infrastructure and policy changes that sustain the ability to bill for telehealth services, which could help increase access to mental health services.
